# Spatiotemporal Variation in Dissolved, Bioavailable, and Particulate Elements and the Abundance of Harmful Algae in Grand Lake

**DOI:** 10.1021/acsestwater.4c00575

**Published:** 2024-11-27

**Authors:** Yetkin Ipek, Parna Ghosh, William E. Mausbach, Punidan D. Jeyasingh

**Affiliations:** † Department of Integrative Biology, Oklahoma State University, Stillwater, Oklahoma 74078, United States; ‡ Grand River Dam Authority, Langley, Oklahoma 74350, United States

**Keywords:** atom-first biology, community ecology, ecological stoichiometry, elemental biology, eutrophication, harmful algal blooms, ionome, nuisance algae

## Abstract

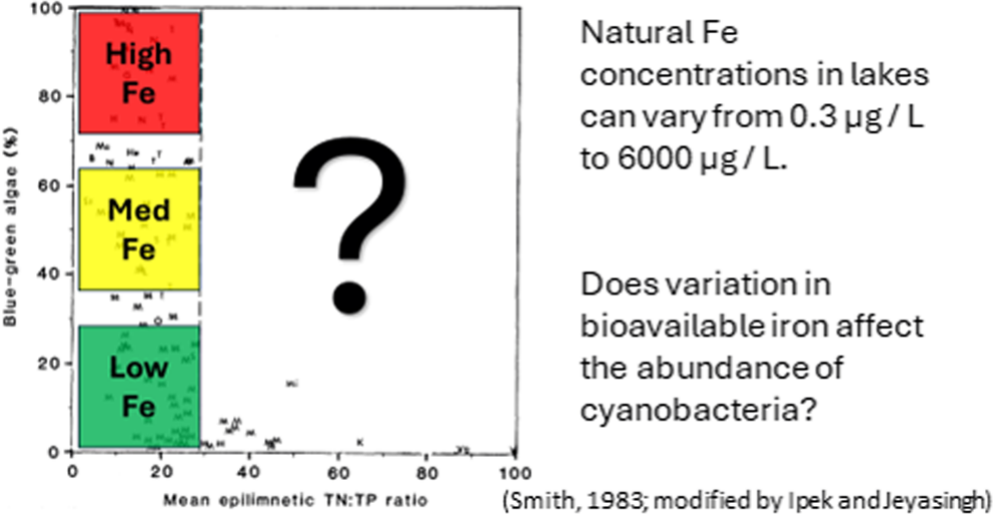

Harmful algal blooms (HABs) are often linked to the increased loading of limiting nutrients such as nitrogen and phosphorus. Little is known about the relevance of other biogenic elements, the supplies of which are spatiotemporally heterogeneous, on HABs. We measured the dissolved, bioavailable, and particulate concentrations of 26 elements at four locations draining different catchments of a large reservoir during three seasons, in addition to the total abundance of phytoplankton and % of cyanobacteria. Finally, we manipulated a key element (Fe) in microcosms to test its effect on the community. Phytoplankton abundance and community structure varied spatiotemporally, with minimal variation in N/P. The variation in environmental supplies of several other elements was correlated with phytoplankton abundance, as well as up to 3 orders of magnitude differences in cyanobacterial yield. Bioassays manipulating Fe impacted total phytoplankton as well as the abundance of cyanobacteria, with Fe-chelated treatments resulting in a significant decline in phytoplankton as well as cyanobacterial yield. In summary, we found substantial heterogeneity in elemental supplies that are relevant to the phytoplankton community. Exploring the relevance of the entire system of elements in the context of HABs may be more rewarding than studies emphasizing a subset of elements.

## Abstract 2

There is limited knowledge of how the spatial and temporal heterogeneity of trace metals affect the growth and community structure of phytoplankton. This study reports a multivariate approach to analyzing the processing of biogenic elements by bloom-forming taxa.

## 1 Introduction

Predicting the abundance and distribution of taxa is a long-standing goal in ecology.^1^ Such ecological approaches are being used to forecast species distributions in the Anthropocene. Harmful algal blooms (HABs), caused by overabundance of certain phytoplankton, are an ever-increasing environmental problem that affects water quality worldwide. The toxins produced by HABs, including neurotoxins, hepatotoxins, cytotoxins, and endotoxins,^2^ affect both human health and ecosystem services such as fisheries and tourism.^3,4^ The annual costs of HAB mitigation are up to $2b in the United States, which is a direct result of such detrimental effects.^5^

While there are several environmental factors that affect HAB formation (e.g., temperature,^6^ pCO_2_,^7^ light intensity^8^), the most common factor thought to promote HABs are limiting nutrients.^9,10^ Nutrients that have enjoyed much of the attention regarding prediction and management of HABs are nitrogen (N) and phosphorus (P), specifically their relative supplies.^11^ This focus is not surprising due to the roles of P^12^ and N^13^ in protein production to support high growth rates. Specifically for cyanobacterial harmful algal blooms (cHABs), an N:P ratio by mass below 22 to 29 is thought to enhance their abundance^11,14^ because N-fixing cyanobacteria can obtain atmospheric N to make up for the limitation in dissolved supply, thus gaining a competitive advantage over other phytoplankton taxa.^15,16^ Despite the fundamental approach based on the growth-promoting effects of these two elements, forecasting HABs by measuring supply N:P still does not provide accurate predictions.^17^

In addition to N and P, there are other essential elements (i.e., trace metals) that take part in the protein structure. These elements play important roles as cofactors in enzymatic reactions of phytoplankton, forming the catalytic centers of proteins.^18,19^ For cyanobacteria, one of the more important trace metals is iron (Fe). While Fe is an important trace metal for all phytoplankton due to its properties as an electron acceptor in the chlorophyll photosystems (PS), Fe is also utilized in the enzyme nitrogenase, which has a dimer structure consisting of Fe and MoFe cofactors that facilitate the binding of atmospheric N to nitrogenase in N_2_ fixation.^20,21^ Other trace metals also affect the cellular functions of proteins and the subsequent growth of phytoplankton based on their environmental supply and availability. Similar to Fe, molybdenum (Mo) is also utilized in the structure of nitrogenase, specifically at the catalytic binding center of MoFe cofactor to facilitate the fixation of atmospheric N_2._^21^ Copper (Cu) takes part in Fe assimilation of phytoplankton through a high-affinity uptake pathway that involves the complexation of multicopper oxidase with Fe permease.^22,23^ Cu-rich nitrite reductase molecules were also previously identified to take part in the nitrate reduction pathways of denitrifying bacteria.^24^ Zinc (Zn) takes part in the structure of carbonic anhydrase enzyme that catalyzes the inorganic carbon acquisition by phytoplankton, resulting in a high Zn demand in near-surface phytoplankton.^18^ Also utilized in the electron transport chains of photosystems, manganese (Mn) is essential in the structure of the water oxidizing complex that facilitates the oxidation of water.^25^ Essential to multiple catalytic processes in phytoplankton (i.e., N fixation, denitrification, and photosynthesis), the processing of trace metals (as cellular demand as a function of environmental supplies) is prone to influence the growth of phytoplankton that depends on such biological processes.

While trace metals are biologically indispensable, there is much variation in their environmental supply (e.g., ref (26)) and bioavailability (e.g., ref (27)), which are further affected by anthropogenic activities.^28^ For example, the supply of Fe can display differences up to 1000-fold among lakes.^29−31^ Similarly, other trace metals such as Cu,^32^ Zn,^33^ Mo,^34^ and Mn^35^ have significant variations in their environmental concentration. Such geochemical heterogeneity has the potential to influence protein activity in primary producers.^18^ The differences in trace metal concentrations are also observed between different locations within a single water body if the system is sufficiently large to demonstrate different geochemical characteristics across spatial scales (e.g., refs (35,36)). Such characteristics indicate that freshwater systems demonstrate vastly different trace metal concentrations at different locations, even with similar supplies of N and P.

Growing system-wide evidence clearly indicates that the supply of any given element affects the demand and processing of multiple elements that make up the organismal biomass (the ionome ref (37)). Studies on phytoplankton have also pointed to simultaneous changes of multiple elements and striking ionome-wide rearrangements in response to a change in the supply of a single element. Altering the supply of P to the green alga *Scenedesmus* resulted in such ionomic changes,^38^ while manipulations to the Fe supply also displayed similar adjustments in the ionome.^39^ Furthermore, Ipek and Jeyasingh^40^ altered the supply N:P as well as Fe to the cyanobacterium *Dolichospermum* and found that Fe supply significantly affected the yield as well as the ionome under N-limited conditions. While several other studies also tested for the effects of trace metal supplies on phytoplankton yield and HAB formation (e.g., refs (41,42)), these studies do not pay attention to the correlated changes in the processing of elements across the ionome and its growth relevance (reviewed in ref (43)). Understanding the association between the supply of the 25-odd biogenic elements and the demand for these elements in phytoplankton ionomes may be the key to better predict and manage HABs.^40^ However, to the best of our knowledge, there are no studies that document the association between the entire suite of biogenic elements and the abundance of harmful algae under natural conditions.

The overarching objective of this study was to test the relevance of metals in impacting standing algal/bacterial biomass and community structure (i.e., ratio of greens to blue-greens) and the extent to which metal availability is spatiotemporally heterogeneous in a large artificial freshwater lake, draining catchments differing in geochemistry and land use. As such, we quantified suites of elements in dissolved, bioavailable, and particulate phases, while testing for associations between elemental abundance and coarse algal community structure (i.e., proportion of cyanobacteria) of Grand Lake o’ the Cherokees, Oklahoma. We utilized the natural environmental concentrations of different sites on Grand Lake that change across a year (temporal) and based on the geochemical and anthropogenic differences between each site (spatial). We hypothesized that while primary production (i.e., standing chlorophyll) would increase with higher concentrations of total N and P measured at each site, phytoplankton community structure would be biased toward cyanobacteria (over green algae) under high P (low N:P) conditions,^11^*and* trace metals (notably Fe and Mo) over other sites. We expected a positive correlation between cellular quotas of elements and productivity and higher metal quotas to be correlated with higher proportions of cyanobacteria in the community. Furthermore, to exemplify potential effects of trace metal supply manipulation, we constructed bioassays to measure relative algal abundances after altering the Fe supply in collected samples from sites. We hypothesized that while overall growth would be dependent on total N and P (and supply N:P) measured at each site, the community structure would be biased toward cyanobacteria over other types of algae under conditions rich in P (low N:P) and trace metals over other sites. Finally, we expected Fe manipulation to impact the yield of all phytoplankton taxa, with increasing Fe availability promoting growth although we predicted larger effects on diazotrophic cyanobacteria.

## 2 Methods

### 2.1 Study Sites

We used the latest watershed modeling report for Grand Lake, Oklahoma,^44^ to identify study sites. Grand Lake forms the boundary between two distinct landscapes (prairie versus Ozark forest), which results in a very heterogeneous watershed. The west side has higher sedimentation than the east side, also with high agricultural runoff. Ozark streams that drain into the lake from the east are high in CaCO_3_. Organic and inorganic loading of N and P from various tributaries into the Grand Lake reservoir indicated up to 100-fold variation. We utilized the differences between N and P concentrations among 4 sites: Horse Creek, Honey Creek, Duck Creek, and Drowning Creek (see Supplementary Figure 1). Cyanobacterial HABs have appeared in Grand Lake since 2011 and are most common in Horse Creek, during midsummer.^45^

### 2.2 Sampling

Each site was sampled in October, March, and June using a Van Dorn Sampler (Eijkelkamp North America, Wilmington, NC), at pelagic (1 m from the water surface) and benthic (1 m from the sediment) depths. Standard water quality parameters and chlorophyll with a probe (EXO2 Sonde, YSI Incorporated, Yellow Springs, OH; calibrated to *R*^2^ > 0.999 for serial dilution of Rhodamine WT solution from 0 to 400 μg/L Chl-a equivalents), and total phytoplankton yield and proportion of cyanobacterial cells (FlowCam Cyano; Yokogawa Fluid Imaging Technologies, Inc., Scarborough, ME; particle size range: 2 μm to 1 mm; Chlorophyll—Ch1:700 nm ±10 nm, Phycocyanin—Ch2:650 nm ±10 nm) were quantified. Finally, DGT (diffusive gradients in thin films) passive samplers (DGT Research, Lancaster, U.K.) were deployed for 48 h to measure the bioavailable concentrations of cationic metals (i.e., Fe, Cu, Zn). Bioavailable concentrations of measured elements through DGT passive samplers were retroactively calculated using deployment time and temperature.^46^

### 2.3 Sample Processing

Water samples were filtered through sterile Whatman cellulose acetate membrane filters (0.45 μm; GE Healthcare Life Sciences, Pittsburgh, PA) to measure the particulate elemental concentrations in phytoplankton. The filtrate from each sample was also subsequently preserved in order to measure the total dissolved concentration of each element. Cellulose acetate filters were later dried in a desiccator at room temperature for 7 days. These filters were digested in 800 μL of HNO_3_ and 400 μL of H_2_O_2_ for 72 h and were diluted to 15 mL with ultrapure (Type 1) water. The remaining filtrate of each sample was also acidified with 4% HNO_3_ for analysis. These samples were analyzed with an ICP-OES analyzer (iCAP 7400; Thermo Scientific, Waltham, MA) to quantify the dissolved and particulate concentrations of elements. Multielement external reference standards (CCV Standard 1, CPI International, Santa Rosa, CA) and an in-line Yttrium internal standard (Peak Performance Inorganic Y Standard, CPI International, Santa Rosa, CA) were used to calibrate the ICP-OES and to correct instrument drift or matrix effects, respectively. Filter blank and water blank solutions were also prepared by digesting cellulose acetate filters with no samples in HNO_3_ and H_2_O_2_ and acidifying ultrapure water with 4% HNO_3_, respectively, to calibrate for background concentrations. We omitted elements that were below the limit of detection in any one replicate, resulting in the following elements being used for analyses: Dissolved: Al, As, B, Ba, Ca, Cd, Co, Cr, Cu, Fe, Li, Mg, Mn, Mo, N, Na, Ni, P, Pb, S, Si, V, and Zn; bioavailable: Ca, Co, Cr, Cu, Fe, Mn, Mo, Ni, Pb, S, V, and Zn; particulate: Al, B, Ba, Cd, Co, Cu, Fe, K, Mn, Mo, Ni, P, Pb, V, and Zn (Table S1).

### 2.4 Fe Bioassays

To further explore the role of Fe supply on the abundance of cyanobacteria, as well as other taxonomic groups of phytoplankton, we conducted bioassays in each sample consisting of 3 treatments. Control represented water samples from each site, while Fe fertilization treatment had +100% dissolved Fe as measured by Lind et al.,^26^ and the Fe chelation treatment had 0.02 mg of deferoxamine, a Fe-specific chelator.^47^ Deferoxamine is a natural Fe chelator, found in the soil bacterium *Streptomyces pilosus*, and has a high affinity to soluble Fe. Complexation studies between deferoxamine and Fe(III) indicated a 1:1 binding rate and a 100% formation rate of complexation between Fe (III) and deferoxamine, indicating a complete limitation of bioavailable Fe.^48^ All samples were kept under 45 μmol/s/m^2^ fluorescent light for 48 h and analyzed using a FlowCam for measurements of yield and the relative proportions of phytoplankton taxa.

### 2.5 Analyses

The significance of differences between measured phytoplankton yield across spatial and temporal scales was tested using a one-way ANOVA as well as Tukey’s tests for pairwise comparisons. The same methods were also used to test the differences in phytoplankton yield across the 3 aforementioned bioassay treatments. Bioavailable concentrations of measured elements through DGT passive samplers were retroactively calculated through regression calculations using deployment time and temperature, as provided by DGT Research.^46^

For site-specific comparisons of elemental profiles, isometric log ratios (ILRs) were constructed. Raw elemental data were subdivided into pairwise linearly independent ratios by splitting the data set into smaller parts. ILRs were calculated as
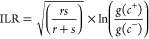
where r and s represent the number of elements on the left- and right-hand side of the ratio and g(c+) and g(c−) minus represent the geometric mean of elemental percentages on the left- and right-hand side of the ratio, respectively.^49^ As ILR ordinations can be difficult to interpret, we examined imbalances for individual elements between sampling sites through elemental imbalance ratios by dividing the measured concentrations of dissolved, bioavailable, and particulate elements at each site to measurements taken from Horse Creek Cove. This comparison was plotted on bar graphs in order to visualize a comparison of each site to growth-optimal conditions of Horse Creek, which was displayed as *x* = 1 reference line on each bar graph. 95% confidence intervals were added to each bar, indicating significant differences whenever there was no overlap observed between the error bars and the reference line.

To better visualize the per-biomass change of each element in comparison to the environmental supply, a version of trophic stoichiometric ratios (TSRs) was constructed as (X_phytoplankton_/yield)/X_water_, where X_phytoplankton_ and X_water_ are the content of each measured element in algal biomass and the water column, respectively.^50^ Please note that chlorophyll-a fluorescence was used as a predictor for the yield, and both dissolved and bioavailable concentrations were used as “X_water_” to calculate different TSRs for each type of measurement. A high TSR indicates a mismatch between the consumer and the environmental supply for any given element per biomass, indicating a high uptake in comparison to the source. To visualize the correlated changes in TSRs between each element and phytoplankton growth, PCAs were constructed to visualize such source-related changes.

## 3 Results

**Figure 1:**
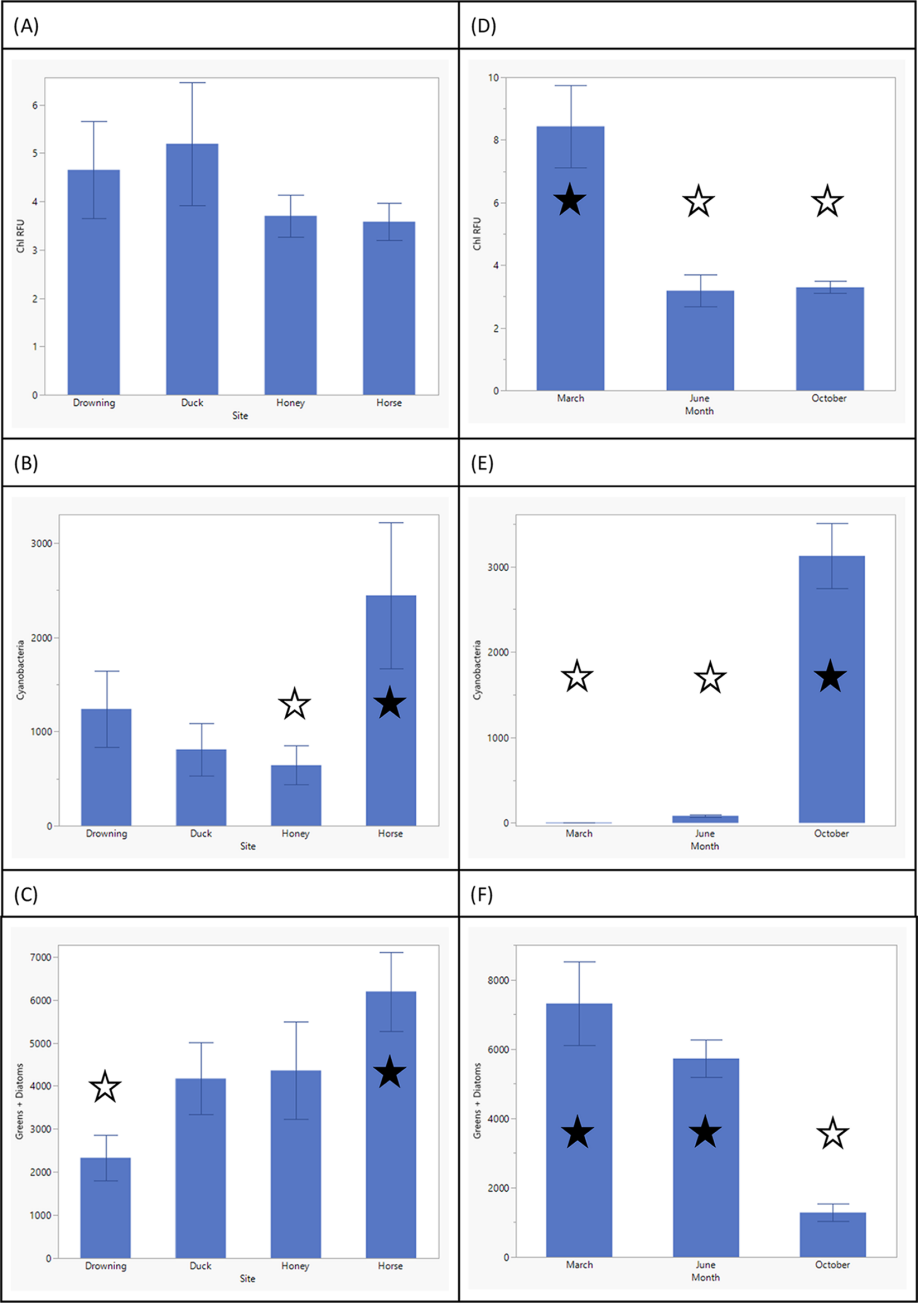
Bar graphs indicating site-specific (A) mean chlorophyll fluorescence (*F*_3, 56_ = 0.80; *P* = 0.49), (B) particle counts for cyanobacterial colonies (*F*_3, 56_ = 2.99; *P* = 0.03), and (C) pooled particle counts for green algae and diatoms (*F*_3, 56_ = 3.21; *P* = 0.02); month-specific (D) mean chlorophyll fluorescence (*F*_2, 57_ = 18.83; *P* < 0.0001), (E) particle counts for cyanobacterial colonies (*F*_2, 57_ = 48.62; *P* < 0.0001), and (F) pooled particle counts for green algae and diatoms (*F*_2, 57_ = 27.48; *P* < 0.0001). Error bars indicate standard error. Tukey pairwise testing indicated significant differences in (B) cyanobacteria counts between Horse Creek and Honey Creek sites (*p* = 0.04) and (C) differences in other algae between Horse Creek and Drowning Creek sites (*p* = 0.01). Chlorophyll fluorescence was significantly different between (D) the month of March and other sampling seasons (*P* < 0.0001). Between different seasons, colony counts for both (E) cyanobacteria and (F) other phytoplankton were significantly different between October and other seasons (*p* < 0.0001). Significant differences between sites and months by Tukey pairwise testing are marked with a star on each plot.

Both spatial (Figure 1A–C) and temporal (Figure 1D–F) differences were significant in phytoplankton abundance as well as community structure. While chlorophyll fluorescence indicated significant temporal differences only between March and other months (Figure 1D), the phytoplankton community structure differed significantly among sites and seasons. Of the 4 sites studied, Horse Creek had the highest abundance of cyanobacteria (Figure 1B) and other phytoplankton (i.e., green algae, diatoms; Figure 1C). Furthermore, between the 3 months of measurements, the month of October favored cyanobacterial yield the most (Figure 1E), whereas the month of March had higher colony counts for other phytoplankton (Figure 1F).

**Figure 2:**
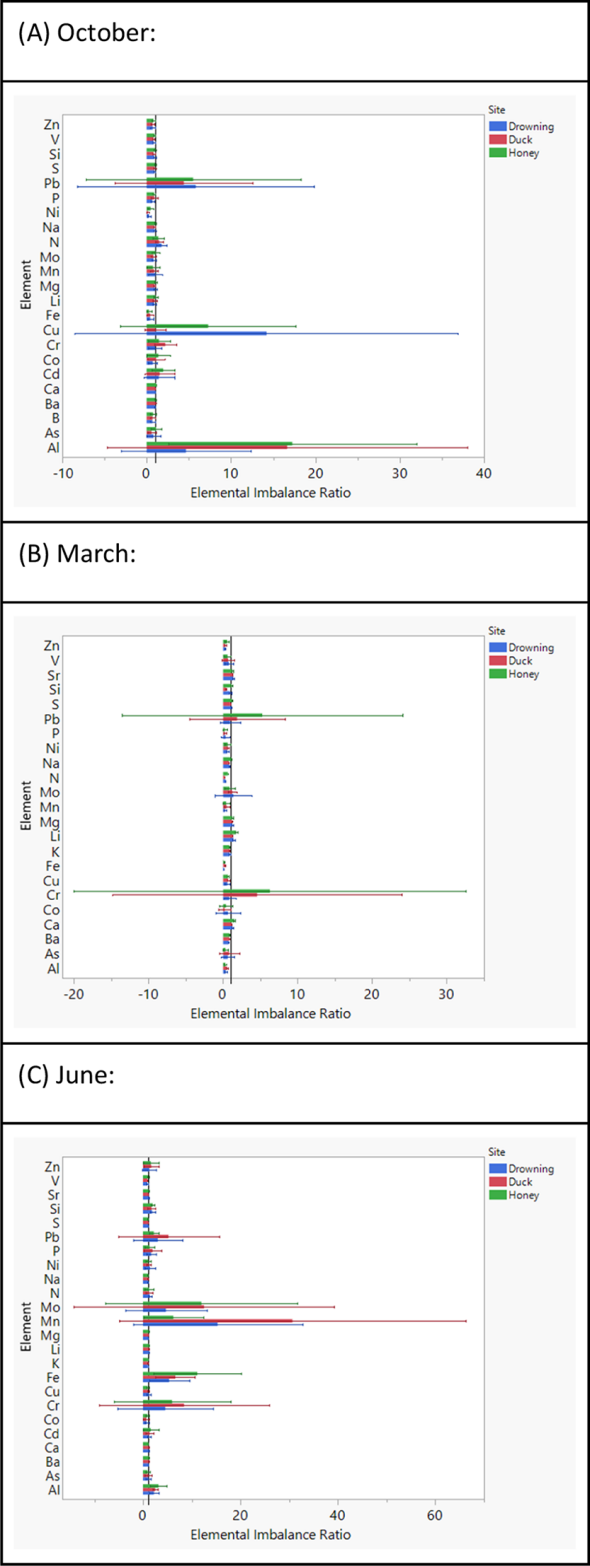
Elemental imbalance ratios comparing the measured dissolved elemental concentrations between different sites to Horse Creek (see reference line, *x* = 1). Each month of measurement (October, March, June) is represented on a separate graph (A, B, C). Error bars represent 95% confidence intervals, and significant differences are assumed when no overlaps are observed between the error bars and the reference line.

**Figure 3:**
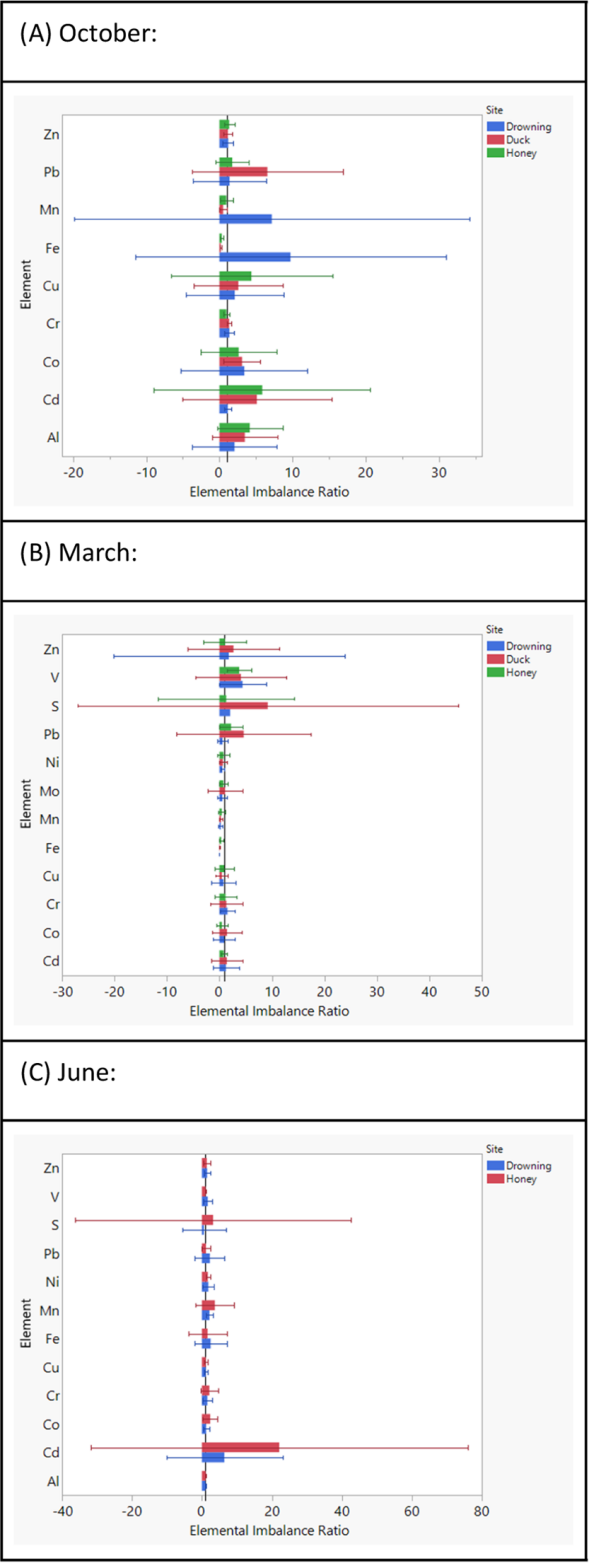
Elemental imbalance ratios comparing the measured bioavailable elemental concentrations between different sites to Horse Creek (see reference line, *x* = 1). Each month of measurement (October, March, June) is represented on a separate graph (A, B, C). Error bars represent 95% confidence intervals, and significant differences are assumed when no overlaps are observed between the error bars and the reference line.

**Figure 4:**
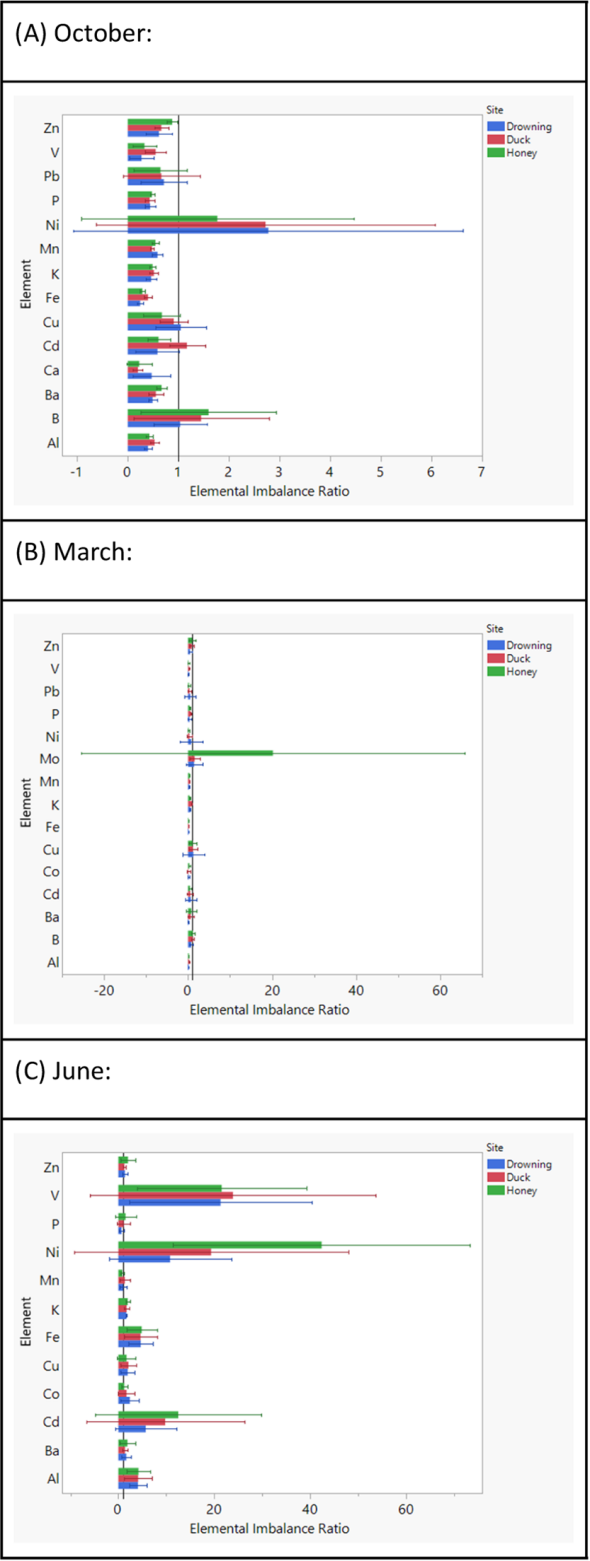
Elemental imbalance ratios comparing the measured particulate elemental concentrations between different sites to Horse Creek (see reference line, *x* = 1). Each month of measurement (October, March, June) is represented on a separate graph (A, B, C). Error bars represent 95% confidence intervals, and significant differences are assumed when no overlaps are observed between the error bars and the reference line.

Since growth conditions appear to be ideal for cyanobacteria in Horse Creek more than in other locations, we decided to use concentration measurements at Horse Creek as a baseline and compare the other 3 sites (Honey Creek, Duck Creek, Drowning Creek) as ratios to the profiles measured at Horse Creek. Represented as elemental imbalance ratios, the concentration of each element was divided by the average at Horse Creek to show relative abundances across the dissolved, bioavailable, and particulate profiles (Figures 2–4).

Across dissolved concentrations of 23 elements, the month of October showed lower concentrations of Fe and Ni in the other 3 sites compared to Horse Creek. Conversely, metals such as Pb, Cu, and Al had higher concentrations in the 3 other sites. Such trends were also observed for Pb and Cr over the month of March, when several elements (e.g., Zn, P, Ni, N, Mn, Fe, Cu, and Al) were significantly higher in Horse Creek. Lastly, the month of June indicated lower concentrations of several elements in Horse Creek compared to other sites, including Pb, Mo, Mn, Fe, and Cr (Figure 2).

Across bioavailable profiles (Figure 3), the month of October showed lower trends in Fe concentrations in Duck and Honey Creek compared to those in Horse Creek. March sampling also indicated similar trends for Fe and Mn concentration, with higher concentrations of these elements in Horse Creek compared to other measured sites. Finally, June had no significant concentration trends between different sites, across the 12 elements measured during that season.

For particulate quotas (Figure 4), the month of October indicated higher cellular concentrations of several elements, including Zn, V, Pb, P, Mn, K, Fe, Ca, Ba, and Al in Horse Creek compared to those at other sites. Similarly, such trends were also apparent in March, where particulate quotas of V, Pb, P, Ni, Mo, Fe, Co, Cd, and Al were observed to be higher in Horse Creek samples compared to other sites. Conversely, particulate concentrations were lower in Horse Creek for several elements during June, including V, Ni, Fe, Cd, and Al.

**Figure 5:**
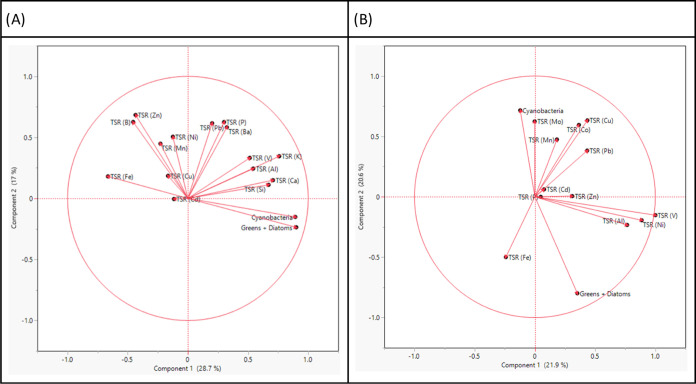
PCA plots representing TSRs of measured elements of filtered algae across all sites and months. See Supplementary Table 2 for loading matrices. TSR values calculated as (*X*_consumer_/Chl RFU)/(X_source_). Calculated TSRs are between filtered algae and (A) total dissolved elements, and (B) bioavailable concentrations of elements as measured by DGT passive samplers to show how much cellular quotas change as a function of availability. Higher TSR scores indicate a greater mismatch between the consumer (algae) and the source. Vectors indicate linear loadings of each element on the two principal component (PC) axes.

To gain a system-wide perspective, we created PCAs on the TSRs of dissolved and bioavailable profiles as well as the two growth vectors for cyanobacteria and other algae (Figure 5). Across dissolved profiles, some of the major cationic metals, such as Fe, Cu, and Zn, had negative loading values on PC1 compared to the yield vectors, indicating a higher retention rate for such elements in lower phytoplankton growth (Figure 5A). In comparison, bioavailable profiles showed inverse loading vectors for cyanobacteria compared to other algae on PC2. The TSR vector for the bioavailable Fe concentration was also negatively correlated to the cyanobacterial yield on PC2, indicating a higher homeostasis for Fe for cyanobacteria at lower growth rates (Figure 5B).

**Figure 6:**
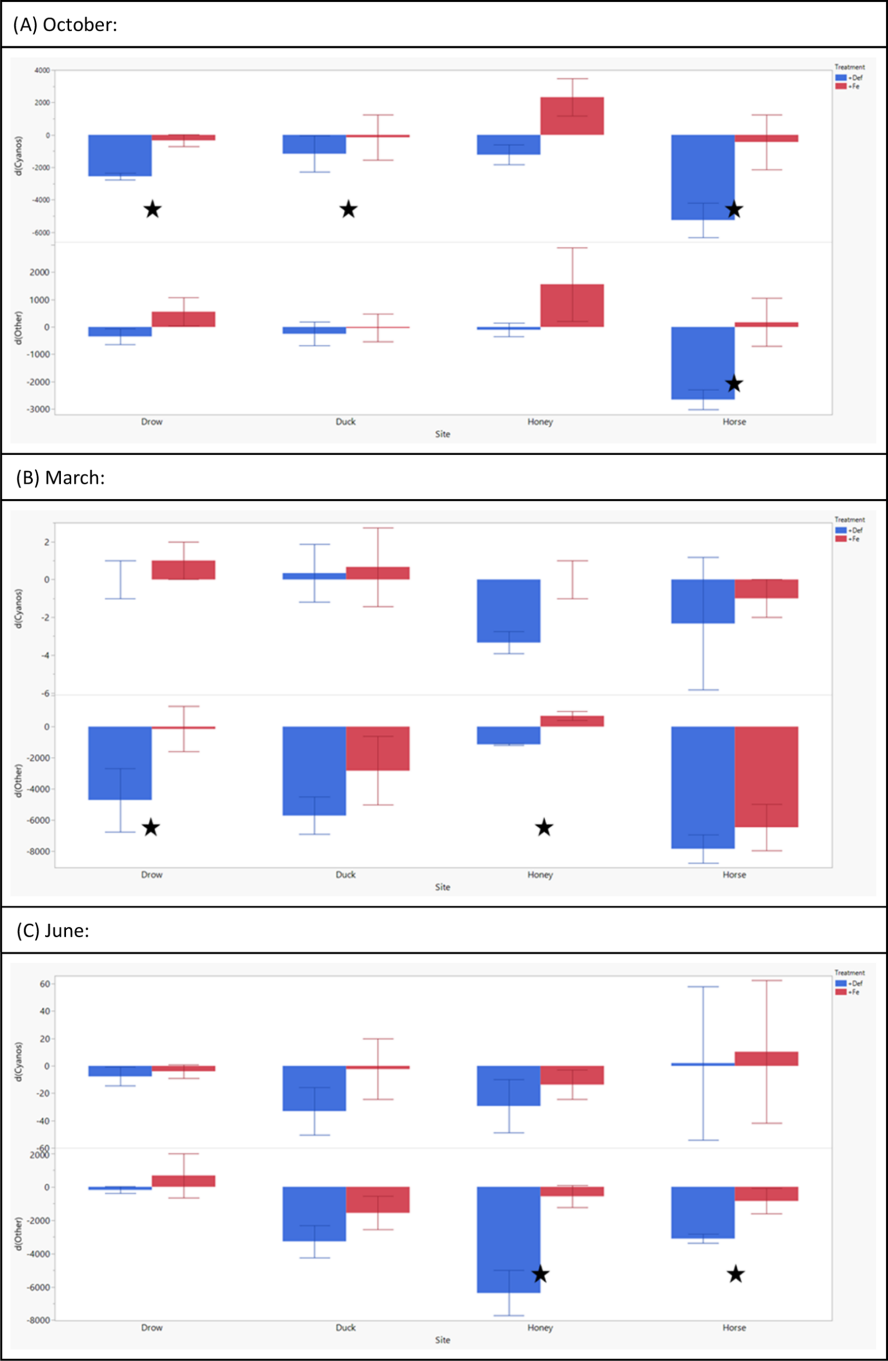
Bar plots showing the net change in the yield of cyanobacteria and other measured algae at the end of the 48 h incubation period. The blue bars indicate the deferoxamine-added treatment, whereas the red bars indicate treatments with added Fe. Error bars represent standard error. Samples collected during each month of sampling are represented on a separate graph for the months of October (A), March (B), and June (C). Significant differences between the two treatments by ANOVA testing are marked with a star on each plot.

Because iron TSRs appeared to be orthogonal to the abundance of cyanobacteria (Figure 5) and prior work regarding the primacy of Fe for freshwater cyanobacteria (e.g., ref (40)), we manipulated Fe supply in bioassays; both through increasing Fe concentrations in gathered samples but also limiting the availability of Fe by chelation through deferoxamine. While Fe-added treatments did not show significant differences compared to control treatments in most cases, colony counts after 48 h of incubation showed significant differences with the addition of deferoxamine for samples gathered in October, March, and June (Figure 6). While both the cyanobacterial yield and the yield of other phytoplankton were significantly affected across sampling sites and months, the strongest effect in the decline of yield was observed in Horse Creek samples in the month of October (Figure 6A).

## 4 Discussion

### 4.1 Spatiotemporal Heterogeneity in Phytoplankton

Both the total abundance and the community structure of phytoplankton are heavily dependent on environmental factors (i.e., temperature,^51,52^ light intensity^8^) that exhibit seasonal as well as geographic differences. Annual shifts in the loading of limiting nutrients remain to be one of the most important drivers of phytoplankton abundance.^52,53^ Limiting nutrients such as N, P, and trace metals are increased in dissolved concentrations through increased inputs into lake water,^54−57^ promoting the growth of green algae mid-to-late spring, along with the increasing temperature as well as light intensity. Green algae are superseded by cyanobacteria in late summer due to their resilience to high temperatures^58−60^ and their ability to vertically migrate to maximize light and nutrient use from different depths in a water body.^61−63^ Our measurements in Grand Lake also showed similar trends. As March sampling indicated the highest chlorophyll fluorescence (Figure 1D), this trend was driven by the abundance of green algae over cyanobacteria in the early spring season (Figure 1E,F). Total chlorophyll fluorescence as well as individual colony counts for all phytoplankton decreased in the early summer. Following this trend, late-season measurements in October showed the highest cyanobacterial yield.

Significant differences in phytoplankton abundance were observed among the sampling sites (Figure S1). While total chlorophyll did not vary among the 4 sampling sites (Figure 1A), the proportion of cyanobacteria did (Figure 1B). As previously observed by Pridmore and Hewitt,^64^ the amount of chlorophyll-a measured per unit of algal cell volume decreased significantly in late summer/early fall seasons compared to early spring. While used as a proxy to measure phytoplankton yield for decades, the cellular concentration of chl-a is also heavily dependent on external factors. Low cellular chl-a concentrations are observed under conditions with nutrient limitation and light saturation.^65,66^ As the summer-fall seasons are characterized by both nutrient depletion and intense light conditions, the observed chl-a compared to the actual colony counts may appear to be lower when representing the phytoplankton yield.^67^

### 4.2 Spatiotemporal Heterogeneity in Dissolved Elements

Horse Creek Cove of Grand Lake has a history of cyanobacterial blooms over the past decade, where cyanobacterial HABs have appeared since 2011. GRDA’s report on average baseline load into Grand Lake estimates the highest flow rate across all sampling sites at 2.93 cm/s. Furthermore, with over 45 kg/day of total P loading and 635 kg/day of total N loading, this site has the highest input of N and P by runoff.^44^ However, as inputs from runoff are insufficient in representing dissolved and bioavailable concentrations of nutrients in lake water, our data compare up to 24 elements across dissolved, bioavailable, and particulate phases. For all measurements, the Horse Creek site was used as a baseline to compare the 3 other sites to visualize the differences in elemental concentrations compared to ideal growth conditions for cyanobacteria. Aside from the correlative effects of nutrient supplies on the growth and community structure of phytoplankton, other environmental variables (temperature, DO, pH, and turbidity) that could alter the phytoplankton yield between the sampling sites were analyzed to look for secondary effects of these parameters (Figure S3). However, no strong heterogeneity in environmental parameters was observed between sites, and the strongest environmental factor that affected the cyanobacterial yield remained to be temperature that only varied temporally.

The comparison of dissolved elements among sites revealed some significant differences, especially regarding cationic metals (Figure 2). During the March sampling, where the rate of nutrient inputs as well as lake mixing are high, Zn, P, Ni, N, Mn, Fe, Cu, and Al were all found to be in significantly higher concentrations in the Horse Creek site compared to other sites. A similar trend was observed for Pb, Mo, Mn, Fe, and Cr in June and for Fe and Ni in October, as Horse Creek had higher concentrations of these elements. As the high N and P concentrations in March allow for high growth rates for phytoplankton,^12,13^ trace metals are also required to support the protein structure and function.^68^ One such element that is consistently in higher concentrations in Horse Creek during all of the 3 sampling seasons is Fe. Fe is important for all phytoplankton as it is a major electron acceptor in photosynthetic machinery. Specifically in cyanobacteria, Fe deficiency has previously resulted in the reduction of chlorophyll levels (chlorosis), as well as the reduction of photosynthetic redox proteins.^69^ More importantly, for cyanobacteria, Fe also has a major role in the process of atmospheric N fixation. As the fixation of N_2_ is facilitated by the enzyme nitrogenase, which has a dimer structure of Fe and MoFe cofactors to bind to N_2_, there is a high demand for cellular Fe to support the function of nitrogenase.^21,70^ Especially in N-deficit conditions, cyanobacterial growth may depend heavily on environmental Fe supply, which allows them to fix atmospheric N_2_ to make up for this limitation.^40^

### 4.3 Spatiotemporal Heterogeneity in Bioavailability of Metals

While the measurements for dissolved concentrations of elements indicated significant differences between trace metal concentrations, measuring the bioavailable concentrations of cationic trace metals was also important to gain an understanding of the relationship between total dissolved nutrient profiles and the amount that was available for cellular use for each element. The bioavailability of cationic metals varies significantly in comparison to total dissolved concentrations based on organic matter complexation (i.e., DOM,^71,72^ siderophores,^73^ redox state,^74,75^ and stratification^76,77^). When the bioavailable concentrations measured by DGT samplers were compared between the 4 sampling sites, Fe was once again observed to be in higher concentrations in Horse Creek during March and October, compared to the 3 other sites (Figure 3). The bioavailability of Fe is particularly important, not only to allow for faster uptake rates to support the function of various proteins^68^ but also due to the binding to DOM,^71^ S,^78^ and/or PO_4_^79^ complexes. More bioavailable Fe might imply sediment release during higher rates of lake mixing,^80^ also allowing for the release of other nutrients that would support higher phytoplankton yield.^78,79^

The variation in the availability resulted in significant differences in particulate quotas of several elements (Figure 4). Phytoplankton from Horse Creek had higher particulate concentrations of P in October and March along with several trace elements including Fe, Ni, Mo, and Zn across sampling seasons, with Fe being consistently higher annually. While higher particulate P was expected to support higher phytoplankton growth in Horse Creek,^12^ higher particulate concentrations of trace metals also indicate a higher demand to support protein structure and functions.^81^ High Fe concentrations in March indicate high phytoplankton abundance along with chlorophyll production to support early season growth.^82^ A similar trend in October also indicates the need for Fe not only in the photosystem but also due to the potential need to produce nitrogenase to fix atmospheric N_2_, along with Mo to support the production of MoFe cofactors of nitrogenase.^21,70^ Following similar trends, Zn is required in phytoplankton for CO_2_ fixation and environmental P acquisition.^81,83^ While such trends indicate potential demand for the aforementioned elements, measuring particulate quotas alone may be misleading, as this approach does not take the environmental supply into account.

### 4.4 Changes in Elemental Processing

Because elemental demand in phytoplankton is highly interrelated and dynamic (e.g., ref (38)), trophic stoichiometric ratios (TSRs^50^) were constructed to normalize the concentration values per-supply and per-biomass. A high TSR value indicates a high concentration for any given element in comparison to the environment and the biomass of the consumer, indicating a mismatch between the environmental supply and the uptake/retention rate of the consumer. A negative relationship between growth vectors and TSR(X) for any given element may indicate a higher homeostatic effect, indicating a higher per-biomass retention rate in comparison to the external supply.^40^ Between dissolved elements, the strongest response to growth limitation was observed for Fe for both cyanobacteria and other types of algae (Figure 5A). Such effects are also observed for B, Zn, Mn, Ni, and Cu. As previously mentioned, Fe is utilized in the photosynthetic machinery as well as the nitrogenase required for N fixation,^21,70^ and Zn takes part in fixing CO_2_ and acquiring environmental P.^81,83^ Cu takes part in the conversion of light into chemical energy in the electron transport chain, whereas Mn also takes part in the photosystem complex.^84,85^ The requirement for these elements in such cellular processes explains the increase of high homeostatic demand (high TSR imbalance) in limited growth conditions. In comparison, calculated TSRs for Si, Ca, Al, K, V, Ba, P, and Pb indicated a positive correlation between growth and elemental uptake. Between these elements, P has a direct correlation with growth rate due to high demand by rRNA to support an increased rate of protein production (Elser et al.).^12^ Si is another element that takes part in phytoplankton growth, specifically in diatoms, due to their silicified cell wall structure.^86^

In comparison to dissolved elemental concentrations, calculated TSRs for bioavailable concentrations of elements indicated a few important differences (Figure 5B). Yield vectors for cyanobacteria and other types of phytoplankton were observed to be inversely correlated. Furthermore, while the TSR vector for Fe again had an inverse relationship with the yield vector for cyanobacteria, there was a close positive correlation between TSR(Fe) and the yield of other types of algae. While this may indicate a stronger homeostatic hoarding of Fe by diazotrophic cyanobacteria compared to green algae and diatoms due to the N fixation metabolism,^21^ the difference in how Fe is processed between total dissolved concentrations and bioavailable concentrations is striking. Fe bioavailability can be altered to become significantly higher for cyanobacteria compared to other phytoplankton by siderophores; compounds with low molecular weight that chelate Fe^3+^ with high affinity are later recovered by cells.^87^ Fe-limited cyanobacteria resort to the increased release of siderophores to increase Fe bioavailability in growth-limited conditions.^69,88^ This negative correlation regarding the processing (i.e., demand/supply) of Fe between cyanobacteria and other phytoplankton taxa is also observed when the elemental TSRs are calculated separately for each site, indicating that site-specific effects are not the reason for this separation (see Supplementary Figure 2). Such a constant trend across all sampling sites is striking, as it points to the difference in the processing of Fe by cyanobacteria compared to the other types of algae to potentially drive changes in community structure in phytoplankton.

It should be noted that an increased demand (or toxicity) from the environmental supply of one element results in correlated changes in the entire network of elements encompassing the ionome. For example, the demand for Fe is mainly regulated by the Fur gene and associated proteins. The complexation of Fe with Fur proteins (in Fe replete conditions) acts as a transcriptional repressor, which further downregulates the production of membrane transport proteins and siderophores.^87,89^ However, when the Fur genes are upregulated, the transport proteins that regulate the uptake of ferrosiderophore complexes can be utilized by other metals to enter phytoplankton cells.^90,91^ Such upregulation mechanisms can also be caused by other factors. For example, Houot et al.^90^ found that Cd stress on phytoplankton downregulates genes that take part in the photosynthetic machinery, resulting in an increased demand for Fe to better tolerate the Cd-induced breakdown of photosystems. Such interelemental correlations point to complex relationships in cellular physiology, where multiple factors need to be considered during the prediction of limiting nutrients.

### 4.5 Bioassays

With the physiological importance of Fe for phytoplankton and with the differences in Fe availability and cellular processing between different taxa, our focus on altering bioavailable Fe allowed us to further underline the significance of such effects. It should be noted that the addition of Fe into collected samples did not result in any significant increases of either phytoplankton yield or the growth of other algae, where trends for *d*_yield_ either resulted in a net decrease in phytoplankton yield or insignificant differences compared to the control treatment. It is important to remember that while Fe is essential for phytoplankton growth, increasing total Fe concentration in a treatment does not result in a linear increase in bioavailable Fe. The additional Fe has a chance to bind to DOM,^71^ S,^78^ and PO_4,_^79^ where the resulting complexes decrease the bioavailability of Fe and other elements such as C, S, and P in the samples. Even with Fe-limited conditions, an increased concentration of Fe can result in the limitation of other elements through the formation of such complexes.

While increasing the Fe concentration did not cause significant changes in phytoplankton yield and community structure, decreasing Fe availability with deferoxamine affected all types of phytoplankton in different scenarios. Over March and June, when the abundance of lake phytoplankton was mainly dominated by green algae and diatoms (i.e., “other” algae), limiting Fe in samples caused a decline in the abundance of these taxa over that of cyanobacteria. Since cyanobacteria had little to no presence in terms of colony counts during these months, Fe limitation likely affects the formation and production of the photosynthetic machinery in all phytoplankton collected in these samples.^69,92^ In contrast, during the month of October, the limitation of Fe mainly affected the cyanobacterial yield over green algae and diatoms, with significant declines only observed for cyanobacterial samples. This also indicates that Fe availability also alters community structures due to the demands of more specific mechanisms. The biggest effect on cyanobacterial yield over other taxa can be attributed to the Fe demand of the N fixation metabolism, where the cellular Fe demand by nitrogenase cannot be met in Fe-limited conditions, especially in low N:P supply.^40,93,94^ While the bioassays utilized deferoxamine, a Fe-specific chelator,^47^ it is important to note that deferoxamine has the potential to bind to other divalent and trivalent metal ions. As a secondary effect following total Fe limitation in these samples, potential colimitation of other essential metals such as Mn, Ni, Cu, and Zn may also be observed as deferoxamine is added to the bioassays.^48^ The potential limitation of metals such as Cu (Fe and O_2_ acquisition, denitrification^22−24^), Zn (C acquisition^18^), Mo (N fixation^21^), and Mn (photosynthesis^25^) affect cellular functions of proteins and the subsequent growth of phytoplankton based on their environmental supply and availability.

### 4.6 Ionomics and Bloom Prediction

Our results are supported by prior studies that demonstrated the effects of trace metal supplies on net and relative abundances of phytoplankton taxa. Vrede and Tranvik^95^ have tested primary production rates in 9 oligotrophic lakes and found that Fe limitation also limited primary production during P-stimulated phytoplankton growth. Kerry et al. have found that while Fe limitation caused decreases in several cyanobacteria species, the rate of Fe scavenging through siderophores was the highest during Fe-rich but N-limited conditions.^96^ Bjorneras et al.^28^ have also underlined the variation in aquatic Fe concentrations as the concentration of Fe was found to have increased in 28% of 340 water bodies in North America and Europe between 1990 and 2013. Downs et al.^41^ conducted micronutrient enrichment bioassays in two New Zealand lakes and found that micronutrient enrichment consisting of B, Co, Cu, and Mo increased primary productivity of a cyanobacterial bloom up to 40% under eutrophic conditions. Finally, Twiss et al.^97^ have observed an increase in picoplankton productivity in samples from Lake Erie, after a micronutrient enrichment containing Fe, Cu, Zn, Co, Mn, and Mo. While these observations highlight the importance of metals in oligotrophic freshwater lakes, our observations in a eutrophic reservoir further add to the growing list of studies reporting associations between metals and HABs.^98,99^

While reporting some striking differences in spatiotemporal heterogeneity of micronutrients and subsequent effects on phytoplankton yield and community structure, our knowledge of the separate effects of these essential nutrients remains largely unielemental. In order to demonstrate the effects of each essential metal on the yield and community structure of freshwater phytoplankton, we rely on predictions based on algal-specific enzymatic processes that each element takes part in (i.e., Fe/Mo and N fixation,^21^ Zn/Mn and photosynthesis^18,25^). As it stands, the literature and past studies on phytoplankton have largely focused on the effects of individual elements and the rates of similar taxon-specific processes. Such approaches to phytoplankton stoichiometry are further limited by the traditional growth models that only account for the effect of one substrate at any given time, compared to “ideal” rates of growth.^100^ As no supplied nutrient functions in isolation, linkages between different elements are vital when assessing system-wide adjustments of multiple physiological pathways in response to the changes in supply of a single limiting element.^101,102^ The complexity in predicting responses to changes in nutrient supplies is multiplied by the heterogeneity of geochemical conditions, where changes in the supplies of nutrients also carry the potential of altering the bioavailability of multiple other elements.^71,78,79^ The complexity behind such phenomena is underlined by the bioassays performed here, as the addition of Fe failed to invoke any taxon-specific growth, and the limitation of Fe through deferoxamine potentially resulted in the colimitation of multiple trace elements.^48^ In that regard, further collection and multivariate analysis of biogeochemical data and physiological responses by phytoplankton are essential in developing a better understanding of HAB formation.^103^

## 5 Conclusions

While the long-standing approaches regarding mitigation of HABs have focused on either single-element (P) or dual-element (N + P) controls,^104−106^ there is evidence that the net growth and the relative abundances of phytoplankton taxa are also controlled by other elements. Moreover, there is heterogeneity in trace metal supplies among freshwater systems.^29−31,95,107^ Our results are consistent with previous observations. The differences in the supply of trace metals, even with similar N/P supplies, can cause significant differences in the growth, community structures, and the processing of multiple elements in the ionomes of phytoplankton, accounting for the dynamics of other elements as functions of supply N/P may be the key to advance our ability to predict and manage HABs. Given the challenges in reliably forecasting and managing HABs and the large unexplored variation in the supply of other biogeochemicals, extending classical approaches (e.g., ref (108)) to gain systems-level understanding as demonstrated here appears to be worthy of effort.

## Data Availability

Data have been submitted as “Spatiotemporal variation in dissolved, bioavailable, and particulate elements and the abundance of harmful algae” (doi:10.5061/dryad.8gtht76vs) to Dryad. The data file can be accessed and downloaded through: https://datadryad.org/stash/share/ahEO_mVJpjT_mpZ8qdECm86npiMlPznOxMSplqE497E

## Supporting Information Available

Sampling site locations, along with values of key physicochemical descriptors (GRDA 2020); PCAs for bioavailable TSR measurements, separated by site (see Figure 5b); PCAs for environmental parameters and colony counts for cyanobacteria and other algae; lists of elements measured for dissolved, bioavailable, and particulate profiles; loading matrices for TSRs (PDF)
