# Supplementary material for: Spatiotemporal Variation in Dissolved, Bioavailable, and Particulate Elements and the Abundance of Harmful Algae in Grand Lake
Source: ACS ES T Water. 2024 Nov 27;4(12):5492–505. doi: 10.1021/acsestwater.4c00575 (PMC11650641; doi:10.1021/acsestwater.4c00575)
Supplement: Supplementary file 1 — ew4c00575_si_001.pdf [file ew4c00575_si_001.pdf]

Title: Spatiotemporal variation in dissolved, bioavailable, and particulate elements and the abundance of harmful algae in Grand Lake

Authors: Yetkin Ipek[1]*, yetkin.ipek@okstate.edu; Parna Ghosh[1], parna.ghosh@okstate.edu; William E. Mausbach[2], william.mausbach@grda.com; Punidan D. Jeyasingh[1], puni.jeyasingh@okstate.edu

[1]Department of Integrative Biology, Oklahoma State University, Stillwater, OK 74078, U.S.
[2]Grand River Dam Authority, Langley, OK 74350, U.S.
*Corresponding author: yetkin.ipek@okstate.edu

Running title: Ecological ionomics of harmful algae

Article type: Environmental Science and Technology – Research Article

Statement of authorship: YI, WM, and PDJ designed the study. YI generated the data with assistance from PG and WM. YI and PDJ analyzed the data and wrote the first draft of the manuscript which was improved by PG and WM.

Data accessibility: Data has been submitted as "Spatiotemporal variation in dissolved, bioavailable, and particulate elements and the abundance of harmful algae" (doi:10.5061/dryad.8gtht76vs) to Dryad. The data file can be accessed and downloaded through:
https://datadryad.org/stash/share/ahEO_mVJpjT_mpZ8qdECm86npiMlPznOxMSplqE497E

Supplementary Figures:

Table 7-7 SWAT Modeled Average Baseline Load into Grand Lake, 2004-2015

Reach ID	Flow (cms)	Sediment (tons/day)	Organic Nitrogen (lb N/day)	Organic Phosphorus (lb P/day)	Nitrate + Nitrite (lb N/day)	Ammonia (lb N/day)	Inorganic Phosphorus (lb P/day)
172 Horse Creek	2.93	44	90	26	1,307	11	85
178 Honey Creek	1.97	23	607	149	561	2	41
182 Duck Creek	0.70	15	18	9	277	1	8
186 Drowning Ck	1.92	13	212	80	937	28	36

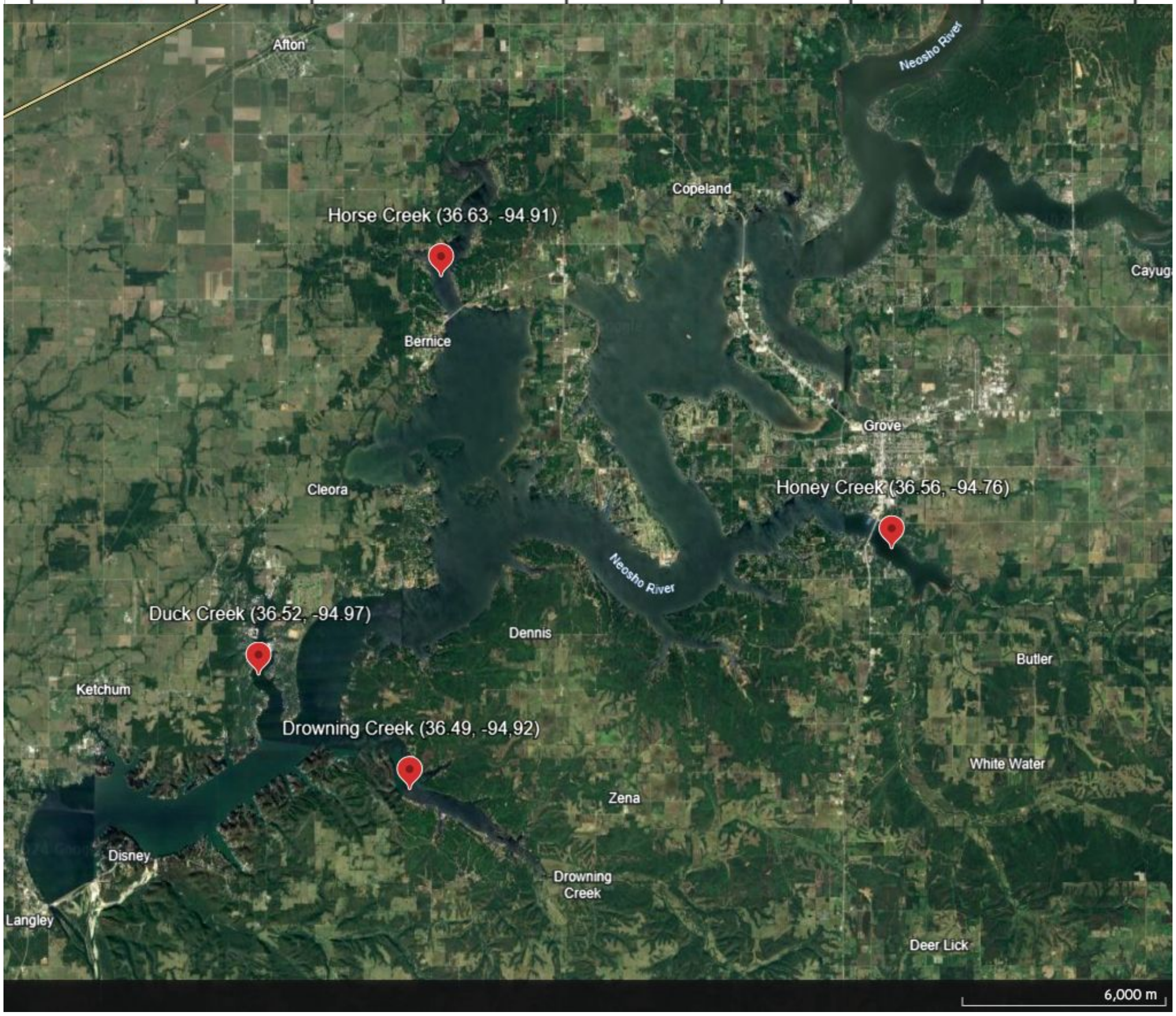

Figure 1: Sampling site locations, along with values of key physicochemical descriptors (GRDA 2020). Geographical coordinates of each site are 36.63241433014393, -94.9144979508068 (Horse Creek); 36.56309289841767, -94.76669227368819 (Honey Creek); 36.56754940310433, -94.76302806339187 (Duck Creek); 36.49063701956669, -94.92981816061665 (Drowning Creek).

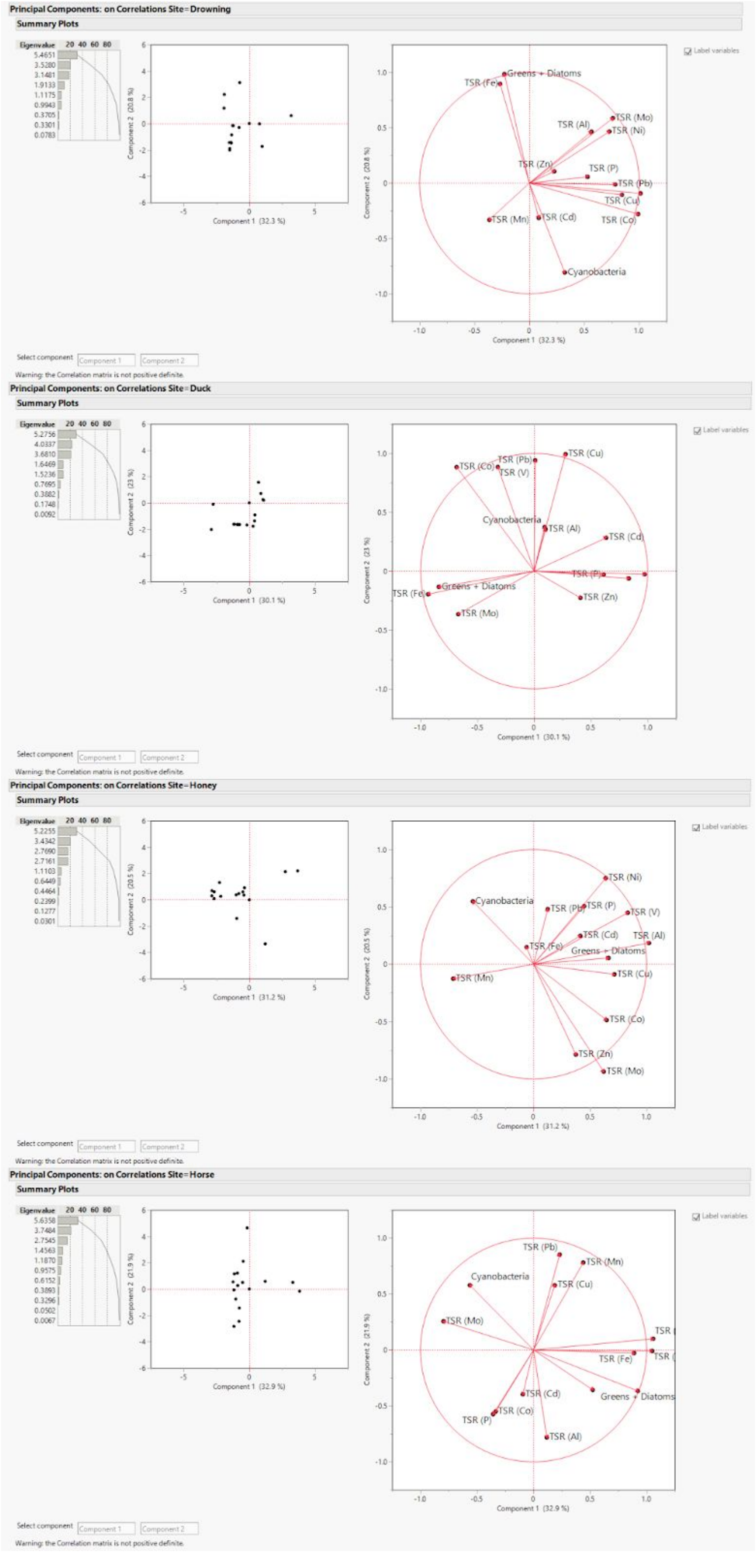

Figure 2: PCAs for bioavailable TSR measurements, separated by site (see Fig. 5b). TSR values calculated as: $(X_{consumer}$ / Chl RFU) / (X_{source}). Higher TSR scores indicate a greater mismatch between the consumer (algae) and the source. Vectors indicate linear loadings of each element on the two principal component (PC) axes.

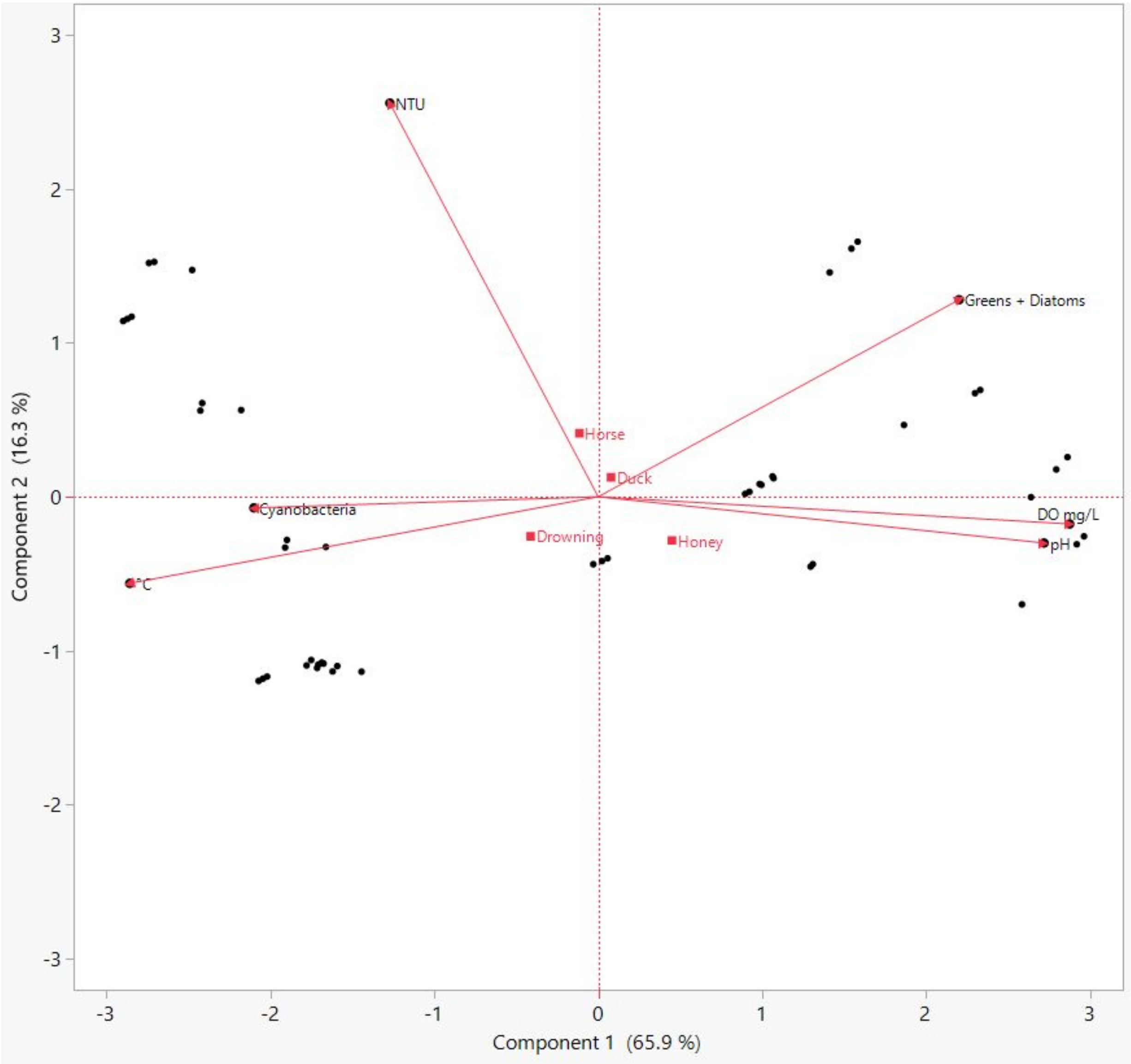

Figure 3: PCAs for environmental parameters and colony counts for cyanobacteria and other algae. Reported environmental parameters include temperature (°C), dissolved oxygen (mg / L), pH, and turbidity (NTU). Vectors indicate linear loadings of each factor on the two principal component (PC) axes.

Table 1: Lists of elements measured for dissolved, bioavailable, and particulate profiles.

Profile	Elements
Dissolved	Al, As, B, Ba, Ca, Cd, Co, Cr, Cu, Fe, Li, Mg, Mn, Mo, N, Na, Ni, P, Pb, S, Si, V, Zn
Bioavailable	Ca, Co, Cr, Cu, Fe, Mn, Mo, Ni, Pb, S, V, Zn
Particulate	Al, B, Ba, Cd, Co, Cu, Fe, K, Mn, Mo, Ni, P, Pb, V, Zn

Table 2: Loading matrices for TSRs (see Fig. 5). A) Loading matrix for dissolved TSR calculations (Fig. 5a). B) Loading matrix for bioavailable TSR calculations (Fig. 5b).

**A**	**Prin1**	**Prin2**
TSR (P)	0.30057	0.62520
TSR (Al)	0.54291	0.24394
TSR (B)	-0.45498	0.62602
TSR (Ba)	0.32391	0.58247
TSR (Ca)	0.70755	0.14934
TSR (Cd)	-0.11604	-0.00474
TSR (Cu)	-0.16554	0.18514
TSR (Fe)	-0.66469	0.18049
TSR (K)	0.75938	0.34588
TSR (Mn)	-0.22843	0.44823
TSR (Ni)	-0.12495	0.50547
TSR (Pb)	0.20155	0.61545
TSR (Si)	0.67054	0.11125
TSR (V)	0.51467	0.33195
TSR (Zn)	-0.43515	0.68328
Cyanobacteria	0.89293	-0.15213
Greens + Diatoms	0.90082	-0.23670

**B**	**Prin1**	**Prin2**
TSR (P)	0.04866	0.00015
TSR (Al)	0.76576	-0.23303
TSR (Cd)	0.07676	0.06187
TSR (Co)	0.36527	0.59501
TSR (Cu)	0.43614	0.63291
TSR (Fe)	-0.24106	-0.49897
TSR (Mo)	-0.00205	0.62389
TSR (Mn)	0.18327	0.47431

B	Prin1	Prin2
TSR (Ni)	0.88722	-0.19437
TSR (Pb)	0.43433	0.38251
TSR (V)	1.00088	-0.15222
TSR (Zn)	0.30994	0.00564
Cyanobacteria	-0.12180	0.71578
Greens + Diatoms	0.35256	-0.79850
